# Supplementary material for: First Detection of Paralytic Shellfish Toxins from Alexandrium pacificum above the Regulatory Limit in Blue Mussels (Mytilus galloprovincialis) in New South Wales, Australia
Source: Microorganisms. 2020 Jun 16;8(6):905. doi: 10.3390/microorganisms8060905 (PMC7356031; doi:10.3390/microorganisms8060905)

Fig. S1A. Standard curve developed from *A. australiense* strain AT-YC-H

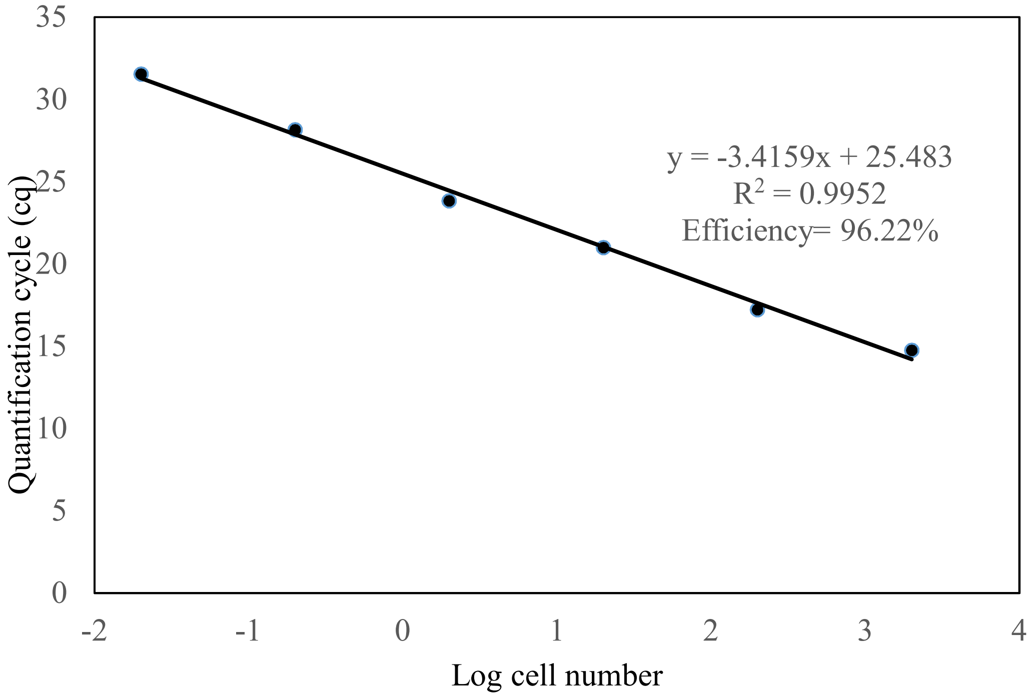

Fig. S1 B. Standard curve developed from *A. pacificum* strain CS300

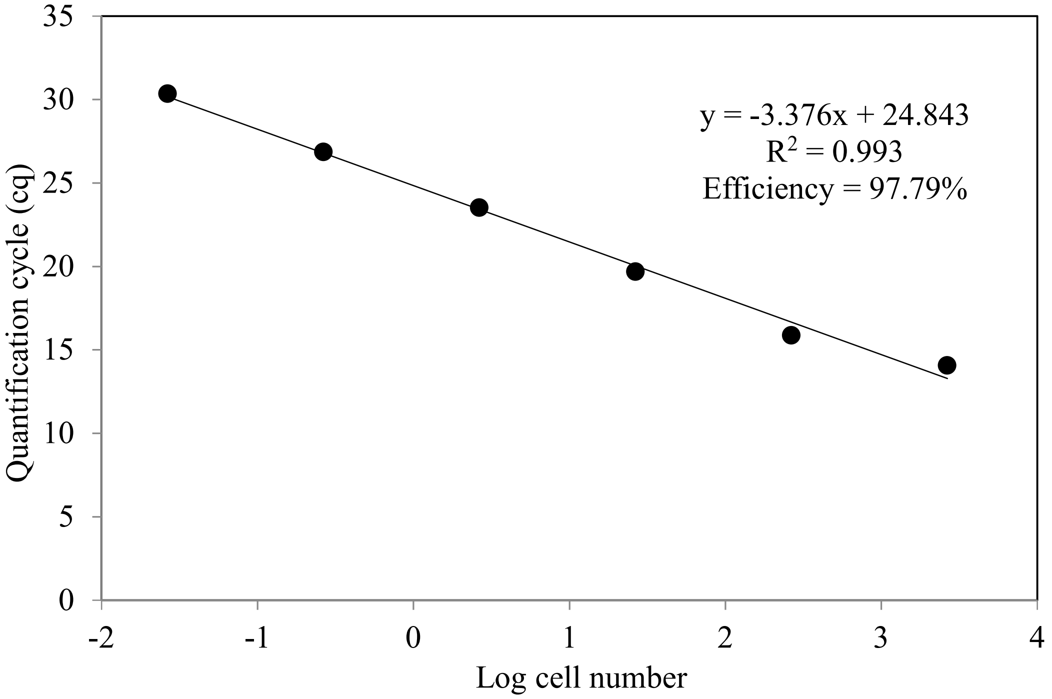

Supplement: Supplementary file 1 [file microorganisms-08-00905-s001.zip › Fig. S1.pdf]
