# Supplementary material for: First Detection of Paralytic Shellfish Toxins from Alexandrium pacificum above the Regulatory Limit in Blue Mussels (Mytilus galloprovincialis) in New South Wales, Australia
Source: Microorganisms. 2020 Jun 16;8(6):905. doi: 10.3390/microorganisms8060905 (PMC7356031; doi:10.3390/microorganisms8060905)
Supplement: Supplementary file 1 [file microorganisms-08-00905-s001.zip › Table S1-2.pdf]

Table S1: Phytoplankton action limits (PALs) for potential PST-producing species in NSW shellfish aquaculture areas. (NSW MBMP, 2016)

Phytoplankton species	Trigger flesh sampling (cells/L)	Alert level – close harvest area pending flesh testing results	Issue public health warning (cells/L)
*Alexandrium minutum**	200	500	5,000
*Alexandrium ostenfeldii**	200	500	5,000
*Alexandrium catenella**	200	500	5,000
*Alexandrium tamarense**	200	500	5,000
Alexandrium spp.*			
Gymnodinium catenatum	1000 mussels<br>2000 other shellfish	5,000	5,000

**Alexandrium* species may be difficult to identify when numbers are low. If any doubt exists, they should be treated as potentially toxic.

Table S2: Procedures for changes to harvest status in classified NSW shellfish harvest areas when potentially harmful phytoplankton species above phytoplankton action limits (PAL) in seawater and algal biotoxins in shellfish flesh are detected. (NSW MBMP, 2016)

Harvest area status	Phytoplankton (cells/L)	Biotoxin (mg/kg)
Open<br>(routine monitoring)	Cells/L < PAL<br>(fortnightly)	Negative result<br>(monthly)
Open<br>(increased monitoring frequency)	Cells/L > sampling PAL<br>(weekly)	Positive result < regulatory limit<br>(weekly)
Closed*	Cells/L > closure PAL<br>or<br>Sample not reported to schedule	Positive result > regulatory limit<br>or<br>Sample not reported to schedule
