# Supplementary material for: First Detection of Paralytic Shellfish Toxins from Alexandrium pacificum above the Regulatory Limit in Blue Mussels (Mytilus galloprovincialis) in New South Wales, Australia
Source: Microorganisms. 2020 Jun 16;8(6):905. doi: 10.3390/microorganisms8060905 (PMC7356031; doi:10.3390/microorganisms8060905)
Supplement: Supplementary file 1 [file microorganisms-08-00905-s001.zip › Table S4.pdf]

Table S4

| **LC-MS Method** | | | |
|---|---|---|---|
| Time | Flow Rate (mL/min) | % A (Water: FA: NH4OH 500:0.075:0.3, V/V/V) | %B (Acetonitrile: water: FA 700:300:0.1, V/V/V) |
| Initial | 0.4 | 2 | 98 |
| 5 | 0.4 | 2 | 98 |
| 7.5 | 0.4 | 50 | 50 |
| 9 | 0.5 | 50 | 50 |
| 9.5 | 0.5 | 2 | 98 |
| 10 | 0.8 | 2 | 98 |
| 10.6 | 0.8 | 2 | 98 |
| 11 | 0.4 | 2 | 98 |
